# Two-Dimensional Covalent Organic Frameworks with Carbazole-Embedded Frameworks Facilitate Photocatalytic and Electrocatalytic Processes

**DOI:** 10.3390/molecules29215071

**Published:** 2024-10-26

**Authors:** Yuchen Xiao, Shanyue Wei, Xiaowei Wu, Canzhong Lu

**Affiliations:** 1 Fujian Institute of Research on the Structure of Matter, Chinese Academy of Sciences, Fuzhou 350002, China; xiaoych@shanghaitech.edu.cn (Y.X.); xmweishanyue@fjirsm.ac.cn (S.W.); 2 Xiamen Key Laboratory of Rare Earth Photoelectric Functional Materials, Institute of Rare Earth Materials, Xiamen 361021, China; 3 Haixi Institutes, Chinese Academy of Sciences, Xiamen 361021, China; 4 School of Physical Science and Technology, ShanghaiTech University, Shanghai 201210, China; 5 Engineering Research Center of Environment-Friendly Function Materials, Ministry of Education, College of Materials Science & Engineering, Huaqiao University, Xiamen 361021, China

**Keywords:** carbazole, covalent organic framework, photocatalysis, oxygen reduction reactions, electrocatalysis, thiazole

## Abstract

Catalytic technologies are pivotal in enhancing energy efficiency, promoting clean energy production, and reducing energy consumption in the chemical industry. The pursuit of novel catalysts for renewable energy is a long-term goal for researchers. In this work, we synthesized three two-dimensional covalent organic frameworks (COFs) featuring electron-rich carbazole-based architectures and evaluated their catalytic performance in photocatalytic organic reactions and electrocatalytic oxygen reduction reactions (ORRs). Pyrene-functionalized COF, termed as FCTD-TAPy, demonstrated excellent photocatalytic performance for amino oxidation coupling and showed a remarkable preference for substrates with electron-withdrawing groups (up to >99% Conv. and >99% Sel). Furthermore, FCTD-TAPy favored a four-electron transfer pathway during the ORR and exhibited favorable reaction kinetics (51.07 mV/dec) and a high turnover frequency (0.011 s^−1^). In contrast, the ORR of benzothiadiazole-based FCTD-TABT favored a two-electron transfer pathway, which exhibited a maximum double-layer capacitance of 14.26 mF cm^−2^, a Tafel slope of 53.01 mV/dec, and a hydrogen peroxide generation rate of 70.3 mmol g^−1^ h^−1^. This work underscores the potential of carbazole-based COFs as advanced catalytic materials and offers new insights into the design of metal-free COFs for enhanced catalytic performance.

## 1. Introduction

The issues of energy shortage [1] and environmental pollution [2] have become increasingly severe, significantly constraining the sustainable development of society. The growing demand for energy and the excessive use of fossil fuels have led to resource depletion and environmental degradation, making the exploration and development of renewable and clean energy essential challenges in the process of societal advancement [3]. In this context, catalytic technologies have emerged as critical tools for achieving efficient and clean energy production. The primary objectives of catalysis are to enhance reaction efficiency, reduce energy consumption, minimize by-product formation, and promote resource recycling [4]. Achieving these goals has driven the continuous development and innovation of novel catalytic materials. In particular, photocatalysis and electrocatalysis have garnered significant attention due to their remarkable potential in energy conversion and environmental protection. Photocatalysis harnesses light energy to drive chemical reactions, such as the photolysis of water to produce hydrogen and the photoreduction of carbon dioxide, effectively converting solar energy into usable chemical energy [5]. Electrocatalysis involves energy-driven catalytic processes, such as in fuel cells and water electrolysis for hydrogen production, with its efficient and environmentally friendly characteristics playing a crucial role in energy storage and conversion [6].

Covalent organic frameworks are crystalline porous polymers constructed from organic building blocks into ordered two- or three-dimensional networks via dynamic covalent bonds [7]. Since Yaghi and colleagues demonstrated the first successful synthesis of COFs in 2005 [8], these materials have garnered extensive research interest and have become one of the fastest-growing fields in materials science [9]. The high porosity, structural tunability, and exceptional stability of COFs render them ideal candidates for catalytic applications [10]. The large surface area and high porosity facilitate the efficient loading of catalytic active sites, selective adsorption, and effective conversion of reactants. The structural tunability allows for the customization of COFs to meet the specific requirements of various catalytic processes. Furthermore, their high thermal and chemical stability enables them to perform effectively under diverse catalytic conditions and ensures a high reuse rate [11,12,13].

As is well known, carbazole-based materials exhibit outstanding photoelectric properties and are extensively utilized in photoelectric devices and organic light-emitting diodes [14,15]. In this work, we designed and synthesized three [4 + 4] two-dimensional covalent organic frameworks (2D-COFs) based on a carbazole structure with sql topology. These COF materials were employed as metal-free catalysts in both photocatalytic amino coupling reactions and electrocatalytic oxygen reduction reactions to evaluate their potential as catalysts.

## 2. Results and Discussion

### 2.1. Syntheses and Characteristics

**Figure 1:**
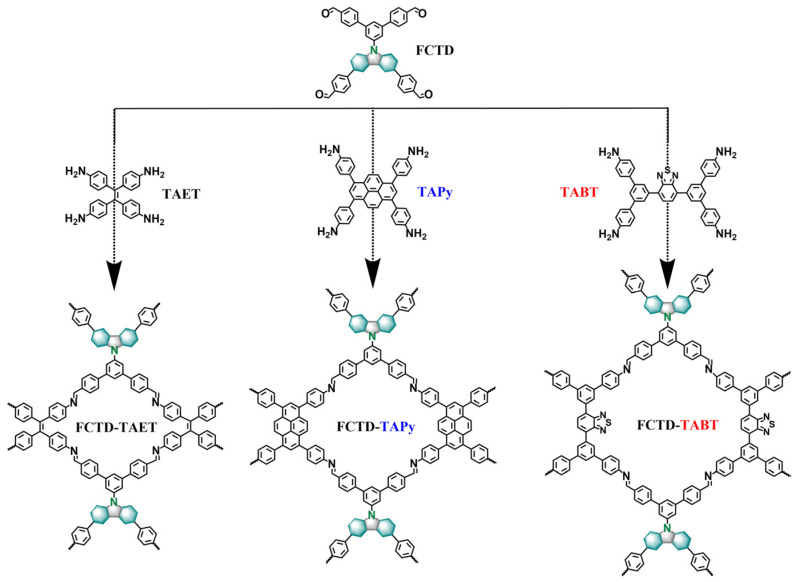
Illustration of the design and synthesis of the COFs.

As illustrated in Figure 1 and detailed in the Experimental Section of the Supplementary Materials, a [4 + 4] condensation reaction was employed to synthesize three COFs. Specifically, these COFs were synthesized by linking FCTD to different building blocks: TAPy, TAET, and TABT under solvothermal conditions. In detail, FCTD-TAPy was synthesized via the Schiff-base reaction of FCTD and TAPy in the presence of 6 mol/L acetic acid, using N, N-dimethylacetamide (DMAC), and n-butyl alcohol (n-BuOH) as solvents at 120 °C for 72 h. FCTD-TAET and FCTD-TABT were synthesized in a similar manner to FCTD-TAPy with variations in the choice of solvent and building blocks.

**Figure 2:**
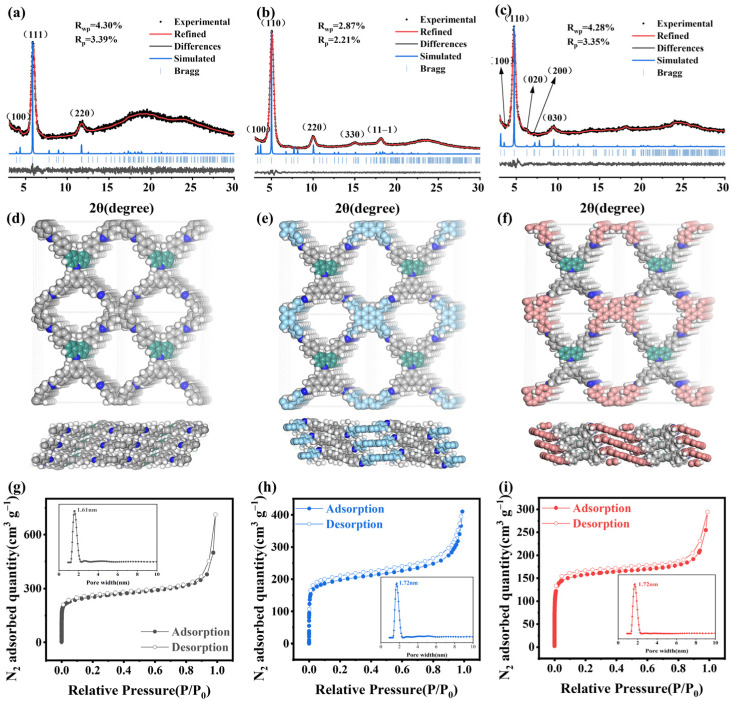
Experimental and simulated PXRD patterns of (**a**) FCTD-TAET, (**b**) FCTD-TAPy, and (**c**) FCTD-TABT; Crystalline structure of (**d**) FCTD-TAET, (**e**) FCTD-TAPy, and (**f**) FCTD-TABT; N_2_ adsorption–desorption isotherms and pore size distribution of (**g**) FCTD-TAET, (**h**) FCTD-TAPy, and (**i**) FCTD-TABT.

The pronounced reflection peaks observed in the powder X-ray diffraction (PXRD) patterns (Figure 2a–c) in the 3° to 10° range, which differ from those of the raw materials, indicate the successful syntheses of crystalline frameworks. The precise periodic structures of FCTD-TAPy, FCTD-TAET, and FCTD-TABT were confirmed through the PXRD analysis in conjunction with structural simulations. Based on the classic C_4_ + C_4_ topology diagram, three 2D sql structural models with the space group of P2 and AA stacking were created (Figure 2d–f) [16,17]. The PXRD results of FCTD-TAPy were additionally fitted by the Pawley refinement as shown in Figure 2b, deducing the refinement lattice parameters of a = 26.740 Å, b = 26.150 Å, c = 5.19 Å, α = γ = 90.0°, and β = 118.0°. The diffraction peaks at 3.73, 5.05, 9.98, 14.93, and 18.11° can be assigned to the (100), (110), (220), (330), and (11-1) facets. The values of Rwp = 2.87% and Rp = 2.21% further attest to the consistency between the theoretical diffraction pattern and experimental PXRD. In the same way, the other two COFs adopt the sql topology (Figure 2a,c). The Pawley refinement yields the unit cell parameters of a = 23.040 Å, b = 22.610 Å, c = 5.44 Å, α = γ = 90.0°, and β = 118° for FCTD-TAET; the diffraction peaks at 4.39, 5.90, and 11.49° can be assigned to the (100), (111), and (220) facets. The Pawley refinement yields the unit cell parameters of a = 27.42 Å, b = 28.16 Å, c =4.46 Å, α = γ = 90.0°, and β = 115° for FCTD-TABT; the diffraction peaks at 3.54, 4.73, 6.28, 7.08, and 9.43° can be assigned to the (100), (111), (020), (200), and (030) facets. An elemental analysis showed that the C/N ratio is close to the theoretical model, which further indicates the rationality of the structural simulation (Table S1).

The porosities of the activated FCTD-TAPy, FCTD-TAET, and FCTD-TABT were evaluated through N_2_ adsorption–desorption isotherms at 77 K (Figure 2g–i). The sorption curves of FCTD-TAPy, FCTD-TAET, and FCTD-TABT were all classified as type Ⅰ isotherms, and the Brunauer–Emmett–Teller (BET) surface areas for them were found to 746.76, 889.56, and 551.33 m^2^/g, respectively. The pore size distributions, as determined from density functional theory (DFT) calculations, revealed primary pore sizes of 1.72 nm for FCTD-TAPy, 1.61 nm for FCTD-TAET, and 1.72 nm for FCTD-TABT. All three COFs exhibited pronounced microporosity.

The chemical structure of the COFs was characterized by Fourier transform infrared (FT-IR) spectroscopy and solid-state nuclear magnetic resonance (SSNMR) spectroscopy. As illustrated in Figure S2, the FT-IR spectra of the COFs exhibit C=N stretching vibration bands at 1621–1623 cm^−1^, indicating the consumption of the starting materials and successful polymerization of the COFs. The solid-state ^13^C NMR spectrum of the COFs displayed a downfield signal at 153–157 ppm, which is attributed to the carbon atom of the C=N bond (Figure S3a–c). The results of FT-IR and ssNMR confirmed that these COFs were successfully condensed via the C=N covalent bonding.

To investigate the thermal stability of the COFs, thermogravimetric analysis (TGA) was conducted under an argon atmosphere. The results (Figure S4) demonstrate that the three COFs exhibit excellent thermal stability, remaining stable up to 500 °C. The chemical stability of the COFs was further confirmed by the retention of characteristic peaks in their PXRD spectra (Figure S5a–c) after immersion in various solvents, including acetonitrile; tetrahydrofuran; N, N-dimethylformamide; 0.1 mol/L NaOH; and 0.1 mol/L HCl for 24 h.

The morphologies of the three COFs were further analyzed by scanning electron microscopy (SEM) and transmission electron microscope (TEM), revealing distinct images of each. FCTD-TAPy exhibits a hollow rod-like structure formed by the agglomeration of irregular spherical particles (Figure S6a,b). FCTD-TAET appears as an irregular cluster of dispersed structures with sizes smaller than 100 nm (Figure S6c,d). In contrast, FCTD-TABT presents a rod-like or folded-sheet structure composed of irregular clumps, also less than 100 nm in size (Figure S6e,f).

**Figure 3:**
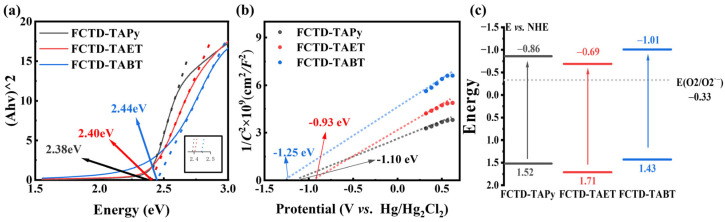
(**a**) Tauc plots, (**b**) Mott–Schottky plots, and (**c**) schematic energy band structures of FCTD-TAPy, FCTD-TAET, and FCTD-TABT.

Solid-state UV/vis absorption spectra were obtained to study the light absorption capacity of these COFs (Figure S7). The results show a broad visible absorption range extending up to visible light (400–800 nm). By using the Kubelka–Munk function, the optical bandgaps of FCTD-TAPy, FCTD-TAET, and FCTD-TABT were determined to be 2.38, 2.40, and 2.44 eV (Figure 3a), respectively. The E_g_ of FCTD-TAPy is the narrowest, which is mainly due to the extended conjugation of the pyrene group. To further investigate the band structures of the three COFs, the conduction band (CB) positions were evaluated using the electrochemical Mott–Schottky measurement method (Figure S8). As shown in Figure 3b, the positive slopes of the M-S plots indicated that these COFs are n-type semiconductors, and the E_CB_ is equal to the flat band potentials for n-type semiconductors. Therefore, the E_CB_ of FCTD-TAPy, FCTD-TAET, and FCTD-TABT are estimated to be−1.10, −0.93, and −1.25 eV (vs. Hg/Hg_2_Cl_2_), respectively. According to the formula E_CB_ = E_VB_−E_g_, we constructed the band structure diagrams for the three COFs (Figure 3c).

The electrostatic potential maps and molecular orbital diagrams of the three COFs were calculated using the density functional theory (DFT). For FCTD-TAPy and FCTD-TAET, the highest occupied molecular orbitals (HOMOs) are located on the electro-donor units TAPy and TAET, while the lowest unoccupied molecular orbital is located in two building blocks (i.e., FCTD and TAPy for FCTD-TAPy, and FCTD and TAET for FCTD-TAET) (Figure S9a–f). In contrast, for FCTD-TABT, the HOMO is primarily located in the FCTD unit, whereas the LUMO is predominantly situated in the TABT unit, which is distinct from the distributions observed in FCTD-TAPy and FCTD-TAET (Figure S9g–i). The HOMO and LUMO of the three COFs did not overlap, indicating that they possess a well-defined donor–acceptor (D–A) structure that promotes electron transfer within the framework.

### 2.2. Photocatalytic Performance Toward Oxidative Coupling of Benzylamines

**Figure 4:**
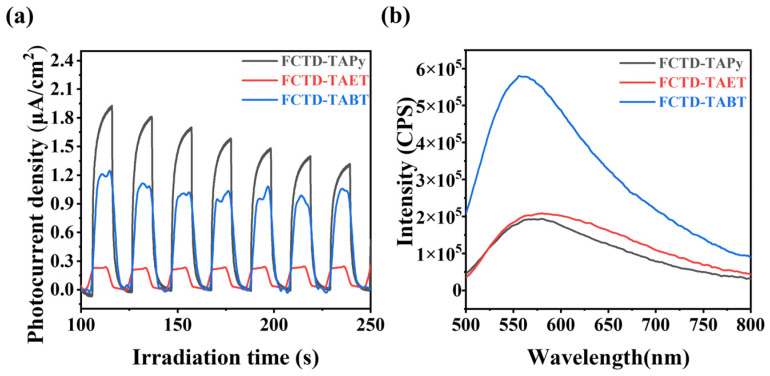
(**a**) Transient photocurrent responses and (**b**) PL spectra of FCTD-TAPy, FCTD-TAET, and CTD-TABT in the solid state.

To evaluate the potential of these COFs as photocatalysts, we conducted a series of tests on their photocatalytic performance [18,19,20]. The transient photocurrent response curves demonstrated that all three COFs possess the capability for photoelectron transfer under illumination. Among them, FCTD-TAPy exhibits the strongest photoelectron transfer capability (Figure 4a). Additionally, the photoluminescence spectra (Figure 4b) revealed that the photoluminescence intensity of FCTD-TAPy was the lowest, suggesting that the photogenerated carrier recombination in FCTD-TAPy was the most subdued, which benefits the photocatalytic reaction.

We investigated the photocatalytic properties of the three COFs under the reaction conditions of using p-methylbenzylamine as the substrate, acetonitrile as the solvent, and blue light irradiation in an oxygen atmosphere. As indicated in entries 1–3 of Table 1, under optimal conditions, the catalytic performance of FCTD-TAPy for the oxidative coupling of p-methylbenzylamine surpasses that of the other two COFs. With FCTD-TAPy as the catalyst, the yield increases progressively with extended reaction times, achieving a nearly complete transformation of the raw material by 12 h. After 12 h of reaction, FCTD-TAPy catalyzed the formation of (*E*)-*N*-(4-methylbenzyl)-1-(p-tolyl)methanimine with a yield of 95% and selectivity of 99%. Comparatively, under the same reaction conditions, the catalytic yields and selectivities for FCTD-TAET and FCTD-TABT were 76% and 99%, and 78% and 78%, respectively.

**Figure 5:**
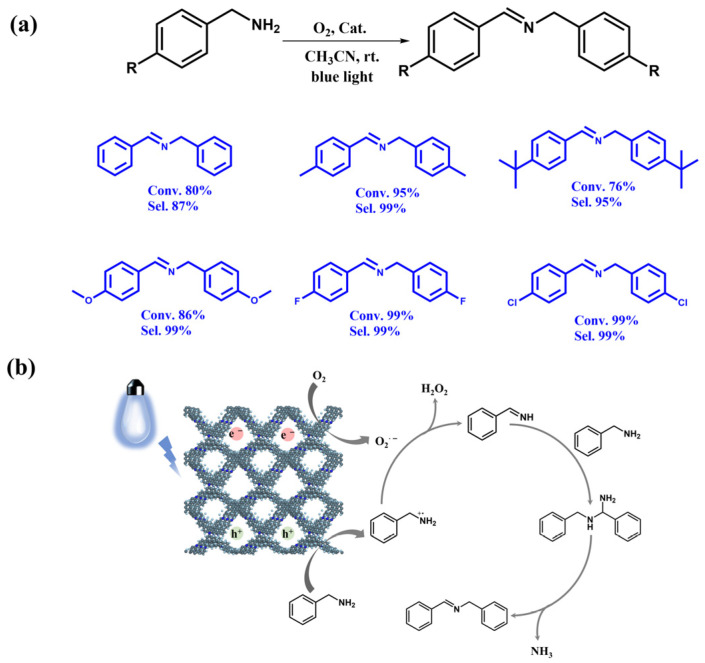
(**a**) The yield and selectivity of photocatalytic selective oxidative coupling of diverse amines, (**b**) Plausible mechanism for the photocatalytic selective oxidative coupling of benzylamine.

To further assess the general applicability of FCTD-TAPy for photocatalytic amino coupling, benzylamine derivatives with various substituents were utilized as reaction substrates (Figure 5a). Under consistent reaction conditions, benzylamine derivatives with electron-withdrawing groups exhibited higher conversion and selectivity (99% conversion and 99% selectivity for -F and -Cl), while those with weak electron-donating groups also showed favorable conversion and selectivity. However, steric hindrance significantly impacted the conversion rate, as demonstrated by the conversion rate of (4-(tert-butyl)phenyl)methanamine, which was only 76%. FCTD-TAPy demonstrated good stability in cyclic experiments with (4-fluorophenyl)methanamine as the substrate. Even after five cycles, FCTD-TAPy was able to maintain a high photocatalytic efficiency, and its crystallinity was largely preserved, as evidenced by PXRD and FT-IR spectra (Figure S10a–c).

To elucidate the intermediate stages and photocatalytic mechanism of the reaction, various trapping agents were incorporated into the reaction for the control tests, and FCTD-TAPy was analyzed using electron paramagnetic resonance (EPR) (Figure S11a–c). The conversion rate plummeted to 5% and 27% when an electron scavenger (AgNO_3_) and a hole scavenger (KI) were introduced, respectively. Additionally, the introduction of a superoxide radical anion (O_2_^·−^) scavenger benzoquinone (BQ) and a radical scavenger hydroquinone (HQ) resulted in a conversion rate of less than 1%. The inclusion of a singlet oxygen scavenger 2,2,6,6-tetramethylpiperidine-1-oxyl led to a reduction in the conversion rate to 55%, and the addition of hydroxyl radical scavenger n-butanol also reduced the conversion rate, albeit slightly, to 88%. In summary, the controlled experiments with various trapping agents indicate that the reaction dynamics are significantly influenced by electrons and holes, with the process being predominantly governed by superoxide radical anions. Screening experiments for necessary conditions and EPR analysis corroborated these findings. In the absence of light, the conversion rate was 46%, and the yield dropped to only 17% without the catalyst (entries 8–9 of Table S2). The EPR spectra revealed that superoxide radical anions could be detected in the dark, albeit with reduced intensity compared to that under illumination (Figure S11c).

Building on these experimental insights and the relevant literature, we proposed a reaction mechanism for the FCTD-TAPy-catalyzed amino oxidative coupling [18,20]. At room temperature, the electrons from the excited state FCTD-TAPy* reduce oxygen to O_2_^·−^, while the benzylamine is oxidized to a benzylamine radical cation by holes, a process enhanced by light exposure. The O_2_^·−^ captures protons from the benzylamine radical cation, forming Ph-CH=NH_2_ and H_2_O_2_. The final target product is produced through a nucleophilic reaction between benzylamine and Ph-CH=NH_2_ (Figure 5b).

### 2.3. Electrocatalytic Oxygen Reduction

**Figure 6:**
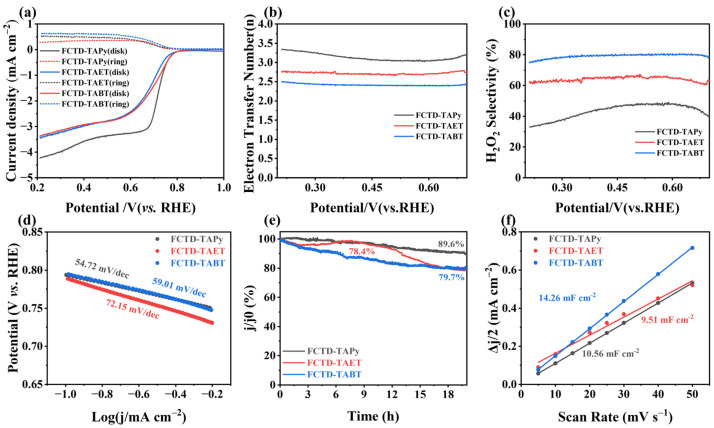
(**a**) LSV curves in O_2_-saturated 0.1 M KOH with a rotating rate of 1600 rpm after 95% iR correction, (**b**) the electron transfer number in 0.2–0.7 V voltage range, (**c**) H_2_O_2_ selectivity in 0.2–0.7 V voltage range, (**d**) Tafel slopes, (**e**) normalized I-t curves at 900 rpm, and (**f**) electrical double-layer capacitor for the three COFs.

We explored their catalytic performance in oxygen reduction reactions (ORRs) [21,22,23]. Cyclic voltammetry (CV) was conducted in both N_2_-saturated and O_2_-saturated KOH (0.1 M) solutions at a scan rate of 50 mV s^−1^ (Figure S12a–c). The CV current density in the O_2_-saturated electrolyte was significantly higher than in the N_2_-saturated electrolyte, with a distinct reduction peak observed at 0.64 V, 0.67 V, and 0.69 V for FCTD-TAPy, FCTD-TAET, and FCTD-TABT, respectively. Their electrocatalytic ORR activity was further assessed using a rotating ring-disk electrode (RRDE) in an O_2_-saturated 0.1 M KOH solution. The linear sweep voltammetry (LSV) curves at 1600 rpm revealed that the onset potentials (E_o_) of FCTD-TAPy, FCTD-TAET, and FCTD-TABT were 0.84, 0.84, and 0.87 V (vs. RHE); half-wave potentials (E_1/2_) were 0.72, 0.69, and 0.68 V (vs. RHE); and the limiting diffusion current densities (*J_L_*) were 4.23, 3.44, and 3.38 mA cm^−2^, respectively (Figure 6a). As indicated in Figure 6d, the Tafel slope for FCTD-TAPy was 51.07 mV/dec, which was lower than 66.55 mV/dec for FCTD-TAET and 53.01 mV/dec for FCTD-TABT. A lower Tafel slope suggested faster ORR kinetics for FCTD-TAPy, which was further corroborated by its highest dynamic current density of 5.92 mA cm^−2^ at 0.7 V (vs. RHE) (Figure S15a). Additionally, the electrical conductivities of the COFs were evaluated via electrochemical impedance spectroscopy. The charge transfer resistances for FCTD-TAPy, FCTD-TAET, and FCTD-TABT were 97, 181, and 207 Ω, respectively (Figure S13), indicating superior electron transfer capabilities in FCTD-TAPy.

The selectivity of these COFs for ORR was determined by RRDE. Between 0.2 V and 0.7 V (vs. RHE), the electron transfer numbers for FCTD-TAPy, FCTD-TAET, and FCTD-TABT ranged from 3.02 to 3.34, 2.65 to 2.79, and 2.38 to 2.50, respectively, while the hydrogen peroxide selectivity were 33–49%, 61–67%, and 75–81%, respectively (Figure 6b,c). The ORR catalyzed by FCTD-TAPy tends toward four-electron transfer, whereas the reactions catalyzed by FCTD-TAET and FCTD-TABT favor two-electron transfers with higher hydrogen peroxide selectivity. The electrochemical active surface area (ECSA), an important indicator of catalytic performance, is proportional to the electrochemical double-layer capacitance (Cdl). The Cdl values were calculated from CV curves at varying sweep speeds (5–50 mV s^−1^) in the voltage range of 1.05–1.15 V (vs. RHE) (Figure S14a–c). As demonstrated in Figure 6f, the Cdl of FCTD-TABT was 14.26 mF cm^−2^, higher than 10.56 mF cm^−2^ for FCTD-TAPy, and 9.51 mF cm^−2^ for FCTD-TAET, suggesting that FCTD-TABT provided more effective active sites and further indicating that the heteroatoms within its structure served as active sites for ORR.

The intrinsic activity of the COFs was evaluated by calculating the turnover frequency (TOF) and mass activity (MA). At 0.7 V (vs. RHE), FCTD-TAPy exhibited the highest TOF and mass activity, with values of 0.01164 s^−1^ and 14.82 A g^−1^, respectively. These metrics underscore its efficiency in promoting O_2_ adsorption and product desorption on the catalyst surface. In comparison, FCTD-TABT and FCTD-TAET demonstrated lower performances with TOF values of 0.00483 s^−1^ and 0.0033 s^−1^, and mass activities of 6.00 A g^−1^ and 4.49 A g^−1^, respectively (Figure S15b).

The long-term catalytic stability of the catalysts was assessed through I-t chronoamperometry (CA) at 0.65 V (vs. RHE). After 20 h of work, FCTD-TAPy maintained 89.6% of its initial current density, indicating superior operational stability. Conversely, FCTD-TAET and FCTD-TABT retained only 78.4% and 79.7% of their initial current densities (Figure 6e). These findings highlight FCTD-TAPy’s working stability. Simultaneously, the concentration of H_2_O_2_ in the electrolyte was quantified using cerium sulfate titration (Figure S16), revealing H_2_O_2_ yields of 24.1 mmol g^−1^ h^−1^ for FCTD-TAET and 70.3 mmol g^−1^ h^−1^ for FCTD-TABT, which indicated significant differences in their catalytic processes.

## 3. Materials and Methods

### 3.1. Materials Characterization

All the chemicals are commercially available and used without further purification. PXRD patterns were collected on a Rigaku Miniflex 600 (Rigaku, Tokyo, Japan) using Cu Kα radiation (λ = 1.5418 Å). Fourier transform infrared was measured on a Thermo Nicolet is 50 FT-IR spectrometers (Thermo Fisher, Waltham, MA, USA) with the Universal ATR accessory between the ranges of 4000 to 500 cm^−1^. Liquid state nuclear magnetic resonance spectroscopy was collected on a Bruker Avance III instrument with an AS500 magnet equipped with a cryoprobe (500 MHz) (Bruker, Karlsruhe, Germany). SEM images were collected using a Apreo S LoVac(Thermo Fisher Scientific, Waltham, MA, USA). TEM images were obtained with a Hitachi H-7650 (Hitachi Limited, Tokyo, Japan). N_2_ adsorption and desorption measurements were performed at 77 K using ASAP 2020, Micromeritics Instrument Corp, USA. Pore size distributions and pore volumes were derived from the adsorption isotherms. UV-Vis diffuse reflectance spectroscopy spectra were obtained by a UV-Vis spectrophotometer (UV-Vis-NIR Cary 5000) (Agilent Technologies, Santa Clara, CA, USA) and the data were converted to Kubelka–Munk functions for the band gap extraction. The thermogravimetric analysis was performed using a Mettler-Toledo TGA/DSC 1 (Hitachi Limited, Tokyo, Japan) under flowing argon with a 5 K min^−1^ ramp rate. The samples were heated in a platinum pan (800 °C, 10 °C min^−1^) under argon flux (60 mL min^−1^). Elemental analysis (EA) was performed using an Elemantar Vario EL cube under the CHN and CHNS models (Elementar, Langenselbold, Germany). Photoluminescence spectroscopy: The emission spectrum was measured with a fluorescence spectrometer (FLS980) (Edinburgh Instruments, Livingston, UK), the excitation wavelength was 455 nm, and the emission range was 475–850 nm. Electron paramagnetic resonance (EPR): The signals of the EPR trapping test were recorded on a Bruker EMXplus paramagnetic resonance spectrometer (Bruker, Karlsruhe, Germany), 300W Xe lamp (450–460 nm) as light irradiation at room temperature. The photoinduced electron-hole pairs of COFs were detected with a mixture of samples of photocatalyst (1 mg)/acetonitrile (1 mL) under air atmosphere. The 5,5-dimethyl-pyrroline-N-oxide (DMPO) and 2,2,6,6-tetramethyl-4-piperidone (TEMP) were selected as the trapping agent of the superoxide radical (O_2_^·−^) and single oxygen (^1^O_2_).

### 3.2. Oxidative Coupling of Amino Groups

The photocatalytic experiment was carried out as follows: the photocatalyst (5 mg) was dispersed in 2 mL of CH_3_CN containing 0.1 mmol benzylamines in a quartz reactor (10 mL). After that, the mixture was bubbled with O_2_ for 10 min and the photoreactor was sealed. The 30 W blue light was used as the light source. The reaction temperature was controlled at 298 K by using the cooling water circulation. After the reaction, the solution was collected, centrifuged, and filtered through a 0.22 μm syringe filter to remove catalyst particles. The products in the filtrate were identified by ^1^H NMR spectrum, and the yield and selectivity were analyzed by ^1^H NMR internal standard. Other benzylamine derivatives were also experimentally tested using the same method.

### 3.3. Electrocatalytic Oxygen Reduction

Electrochemical characterization was performed on an RRDE (IPS, Germany) instrumentation equipped with a high-speed and stable rotator and a CHI 760E (Chenhua, Shanghai, China) with a general three-electrode system at the test conditions of 0.1 M KOH at 25 °C. The three-electrode system consisted of a working electrode (glassy carbon electrode supported by catalyst), a reference electrode (calomel electrode), and a counter electrode (Pt). The Nernst equation was used to convert potentials from the reference electrode to the RHE scale. Under the 0.1M KOH testing condition, the three electrodes were as follows: the working electrode was the catalyst loading rotating ring-disk electrode; the reference electrode and the counter electrode were the calibrated saturated calomel electrode (reference electrode had almost no potential difference with a new Hg/Hg_2_Cl_2_ electrode) and platinum mesh electrode, respectively. The uniform catalyst ink was prepared by mixing 5.0 mg COF, 3.0 mg Ketjen black, 265 μL isopropyl alcohol, 215 μL ultrapure water, and 20 μL Nafion (5 wt%, Alfa), which was then ultra-sonicated for 1 h at 20 °C. Then, 7.9 μL of the ink was loaded onto the working electrode, and O_2_ or N_2_ was successively purged into the electrolytic cell with the flow rate of 80 mL/min for 40 min to ensure that the O_2_ or N_2_ in the test environment was saturated. For the rotating ring-disk electrode tests (RRDE, 0.1963 cm^2^ in area, collection coefficient 0.344), linear sweep voltammetry (LSV) was performed in N_2_-saturated and O_2_-saturated 0.1 M KOH at a scan rate of 10 mV/s under 1600 rpm. Cyclic voltammetry (CV) was measured in O_2_-saturated 0.1 M KOH under various scan rates (5, 10, 20, 30, 40, and 50 mV/s). The long-term stability test was conducted by measuring the current changes in the catalyst at a rotating speed of 900 rpm in the O_2_-saturated electrolyte.

## 4. Conclusions

We designed and synthesized three 2D-COFs with sql net (named FCTD-TAPy, FCTD-TAET, and FCTD-TABT). These COFs exhibited suitable energy band structures, good stability, open channels, and photosensitivity. Among them, FCTD-TAPy demonstrated better catalytic activity and cycling stability for the photocatalytic amino oxidation coupling reaction and showed superior catalytic performance for substrates with electron-withdrawing groups. This may be attributed to its weak light-generated carrier recombination, lower LUMO level, and better photoelectric response capability. In addition, imine bonds were recognized as active sites for oxygen reduction reaction (ORR) catalysis. Pyrene-based FCTD-TAPy exhibited a tendency towards the four-electron transfer process during ORR, demonstrating the fastest kinetics and good stability among the three COFs. On the other hand, the benzothiadiazole-based FCTD-TABT tended to favor a two-electron transfer process, showing the highest number of active sites, as well as excellent selectivity and yield for hydrogen peroxide. Overall, this study demonstrates the potential of three new carbazole-based COFs in the field of catalysis and provides new insights for the design of dual functional catalysts capable of photocatalytic and electrocatalytic performances.

## Figures and Tables

**Table 1:** Amino oxidation coupling reaction by using FCTD COFs.

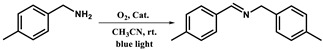				
Entry	Photocatalyst	Time (h)	Conv. (%)	Sel. (%)
1	FCTD-TAPy	12	95	99
2	FCTD-TAET	12	76	99
3	FCTD-TABT	12	78	78
4	FCTD-TAPy	4	55	99
5	FCTD-TAPy	8	85	99

Reaction conditions: substrate (0.1 mmol), photocatalyst (5.0 mg), O_2_ (1 atm), CH_3_CN (2.0 mL), 30 W blue light (λ = 450–460 nm), and room temperature.

## Data Availability

All the data are available within the manuscript. Additional data will be provided upon request from the corresponding authors.

## Supplementary Materials

The following supporting information can be downloaded at https://www.mdpi.com/article/10.3390/molecules29215071/s1, Figure S1: PXRD of COFs; Figure S2: FTIR of COFs; Figure S3: CP/MAS NMR of COFs; Figure S4: TGA of COFs; Figure S5: Stability tests of COFs; Figure S6: SEM images of COFs; Figure S7: UV-Vis spectra of COFs; Figure S8: Mott–Schottky plots of COFs; Figure S9: Electrostatic potential of models for COFs; Figure S10: Reuse tests of COFs; Figure S11: EPR spectra of COFs; Figure S12: CV curves of COFs; Figure S13: Nyquist plots of COFs; Figure S14: CV at different scanning rates of COFs; Figure S15: E_1/2_ and TOFs for COFs; Figure S16: H_2_O_2_ production of COFs; Table S1: Elemental analyses (C, H, and N) for COFs; Table S2: Influence of different trapping agents on catalytic effect; Tables S3–S5: Fractional atomic coordinates for COFs; Table S6: Different COFs’ performance for electrocatalytic ORR. Table S7. Comparisons of different photocatalysts for the selective oxidation of benzylamine. References [18,20,24,25,26,27,28,29,30,31,32,33,34,35,36,37,38] are cited in the Supplementary Materials.
