# Supplementary material for: Two-Dimensional Covalent Organic Frameworks with Carbazole-Embedded Frameworks Facilitate Photocatalytic and Electrocatalytic Processes
Source: Molecules. 2024 Oct 26;29(21):5071. doi: 10.3390/molecules29215071 (PMC11547665; doi:10.3390/molecules29215071)
Supplement: Supplementary file 1 [file molecules-29-05071-s001.zip › molecules-3262200-supplementary.pdf]

*Supporting information for*

# 2D Covalent Organic Frameworks with Carbazole-embedded Frameworks Facilitate Photocatalytic and electrocatalytic Processes

**Yuchen Xiao [1,2,3], Shanyue Wei [1,2,4], Xiaowei Wu [1,2,*] and Can-Zhong Lu [1,2,3,*]**

[1] *State Key Laboratory of Structural Chemistry, Fujian Institute of Research on the Structure of Matter, Chinese Academy of Sciences, Fuzhou 350002, China.*
[2]*Xiamen Key Laboratory of Rare Earth Photoelectric Functional Materials, Xiamen Institute of Rare Earth Materials, Haixi Institutes, Chinese Academy of Sciences, Xiamen, 361021, China.*
[3]*School of Physical Science and Technology, ShanghaiTech University, Shanghai, 201210, China.*
[4]*Engineering Research Center of Environment-Friendly Function Materials, Ministry of Education, College of Materials Science & Engineering, Huaqiao University, Xiamen 361021, China.*

Email: xmwuxiaowei@fjirsm.ac.cn; czlu@fjirsm.ac.cn

## 1. Methods

**Crystal structure modeling**: Structural modeling of COFs was generated using the Materials Studio program (ver2019), the lattice model was geometrically optimized using force-field based method (Forcite, UFF, Ewald summations, $Q_{eq}$). Pawley fitting was conducted by using the Reflex module.

**Density-functional theory simulation**: The electrostatic potential and molecular orbital of COFs were calculated using DFT based on the Materials Studios (MS) software. Considering the limitation of computing resources, the model is established by using FCTD coupled with two amino monomers via imine linkage. GGA-BLYP function of DMol3 module was used for carrying out structural optimization and calculating electrostatic potential and molecular orbital. The electronic energy was considered self-consistent when the energy change was smaller than $10^{-5}$ eV. A geometry optimization was considered convergent when the force change was smaller than 0.02 eV/Å.

**$H_2O_2$ quantification**: The $H_2O_2$ concentration was measured by traditional cerium sulfate $Ce(SO_4)_2$ titration method based on the mechanism that a yellow solution of

$Ce^{4+}$ would be reduced by $H_2O_2$ to colorless $Ce^{3+}$.

$$2Ce^{4+} + H_2O_2 \rightarrow 2Ce^{3+} + 2H^+ + O_2$$

Therefore, the concentration of $H_2O_2$ (M) can be determined by the following equation: $M(H_2O_2) = \frac{1}{2}\Delta M(Ce^{4+})$.

Typically, the yellow transparent $Ce(SO_4)_2$ solution (1 mM) was prepared by dissolving 33.2 mg $Ce(SO_4)_2$ in 100 ml 0.5 M sulfuric acid solution. Then the collected electrolyte (400 μl) was titrated into 3 mL $Ce(SO_4)_2$ solution (1 mM) and shaking the mixed solution for two minutes. As a counterpart, the 400 μL 0.1 M KOH solution were titrated into 3 mL $Ce(SO_4)_2$ solution (1 mM) to obtain the mixed solution unreacted. Based on the linear relationship between the signal intensity of ultraviolet-visible spectroscopy (at around 319 nm) and $Ce^{4+}$ concentration. The concentration of $Ce^{4+}$ before and after the reaction can be measured by ultraviolet-visible spectroscopy. Therefore, the $H_2O_2$ concentrations of the samples could be obtained.

## 2. Synthesis procedures

**5'-(3,6-bis(4-formylphenyl)-9H-carbazol-9-yl)-[1,1':3',1''-terphenyl]-4,4''-dicarbaldehyde (FCTD)**

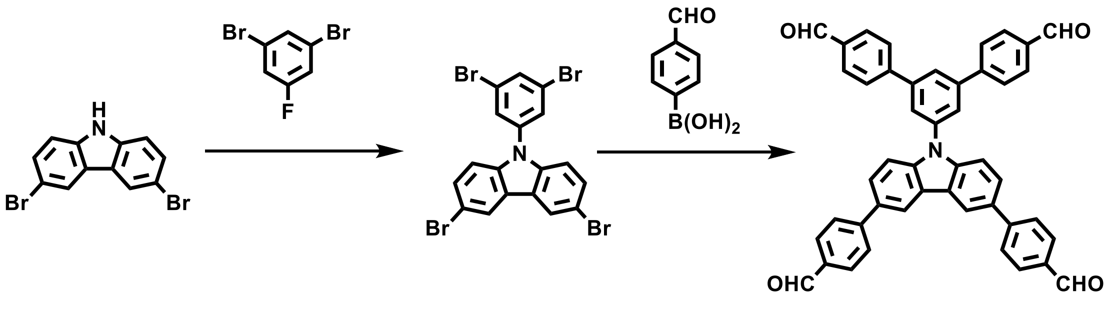


1 FCTD

975 mg (3 mmol, 1 eq.)3,6-dibromo-9H-carbazole, 1.52 mL (3048 mg, 12 mmol, 4 eq.) 1,3-dibromo-5-fluorobenzene, 3910 mg (12 mmol, 4 eq.) $Cs_2CO_3$ and 30 mL N, N-Dimethylformamide were weighed and packed in 100 mL round-bottom flask. The reaction was stirred at 150°C for 24h in air atmosphere. After cooling to room temperature, water is added to precipitate the solid. Filter and wash solids with salt

water and ethyl acetate in turn, drying in vacuum to give a grey powder of **1** (yield: 85%). $^1$H NMR (500 MHz, Chloroform-d) δ 8.21 (d, 2H), 7.83 (t, 1H), 7.66 (d, 2H), 7.57 (dd, 2H), 7.29 (d, 2H).

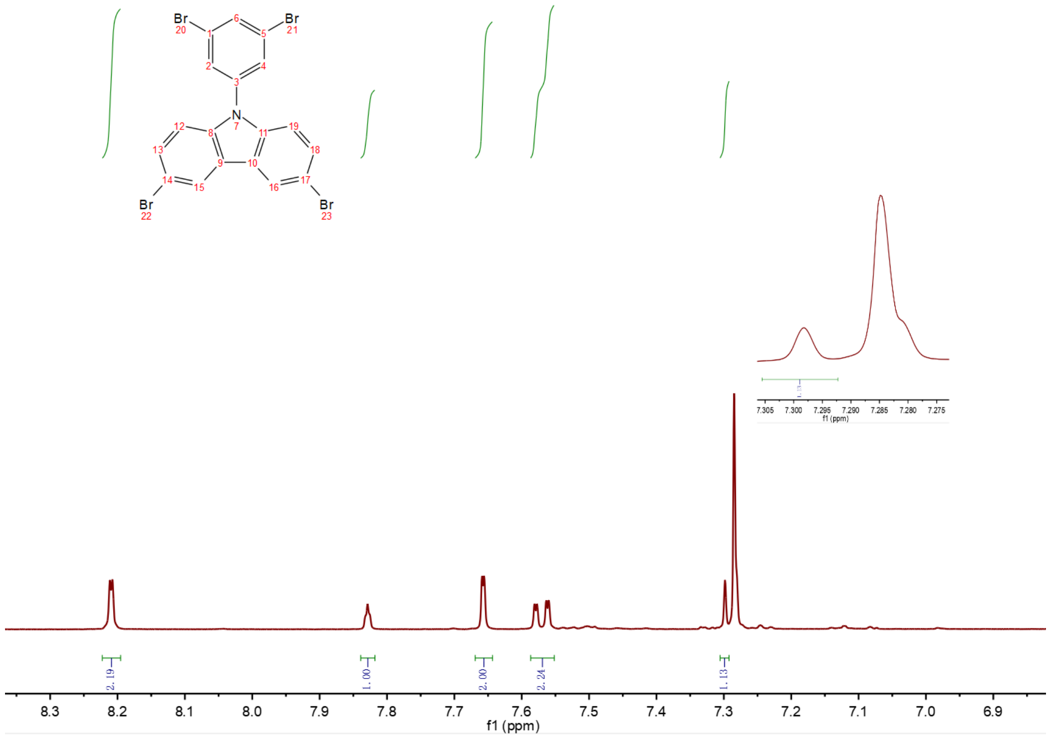


280 mg (0.5 mmol, 1 eq.) **1**, 375 mg (2.5 mmol, 5 eq.) 4-formylphenylboronic acid, 553 mg (4 mmol, 8 eq.) $K_2CO_3$ and 11.6 mg (0.01 mmol, 0,02 eq.) $Pd(PPh_3)_4$ were weighed and packed in 120 mL pressure bottle and adding 15 mL 1,4-dioxane and 3 mL $H_2O$ as solvent. The mixture was vacuumed and filled with inert gas three times and stirred at 120 °C for 24 h. After cooling to room temperature, the mixture was filtrated followed by rinsing with water and alcohol, and drying in vacuum to give 284 mg yellow powder of 5'-(3,6-bis(4-formylphenyl)-9H-carbazol-9-yl)-[1,1':3',1''-terphenyl]-4,4''-dicarbaldehyde (yield: 86%).$^1$H NMR (500 MHz, DMSO-$d_6$) δ 10.13 – 10.06 (m, 4H), 9.02 – 8.88 (m, 2H), 8.38 – 8.34 (s,1H), 8.26 – 7.95 (m, 14H), 7.76 – 7.54 (m, 8H).

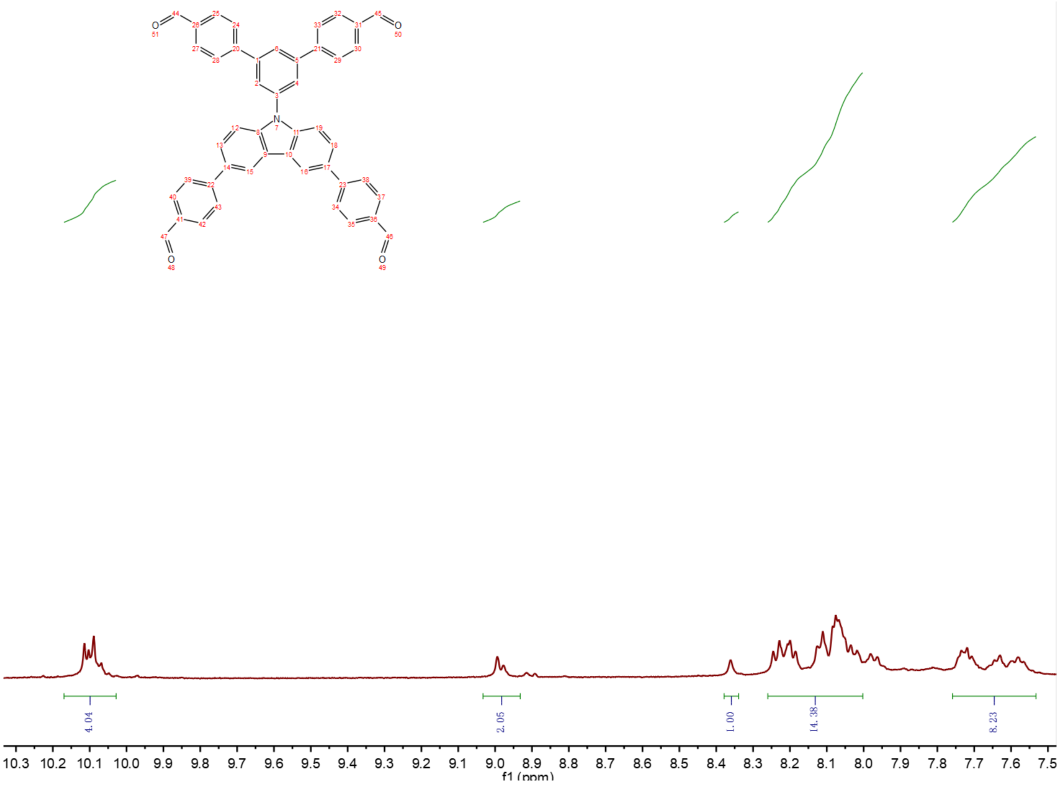

**FCTD-TAPy**

**FCTD** (19.8 mg, 0.03 mmol) and **TAPy** (17.0 mg, 0.03 mmol) were added into a 10 mL Pyrex tube; then, a mixture of N,N-dimethylacetamide (DMAC, 1.0 mL), *n*-butanol (n-BuOH, 1.0 mL), and acetic acid (aq 6 M, 0.2 mL) was added into this Pyrex tube. The Pyrex tube was sealed after being evacuated under vacuum for three pump–thaw cycles in a liquid nitrogen bath. After that, the sealed Pyrex tube was placed in a vacuum oven at 120 °C for 3 days. The solid was isolated by centrifugation, washed with ethyl acetate, and further purified by Soxhlet extraction with tetrahydrofuran, methanol, and trichloromethane. The sample was then transferred to a vacuum chamber and evacuated to 20 mTorr at 50 °C for 24 h, yielding COFs as yellow powders (yield: 33.2 mg, 90.02%).

**FCTD-TAET**

**FCTD** (19.8 mg, 0.03 mmol) and **TAET** (11.8 mg, 0.03 mmol) were added into a 10 mL Pyrex tube; then, a mixture of N,N-dimethylacetamide (DMAC, 1.0 mL), mesitylene(Mes, 1.0 mL), and acetic acid (aq 6 M, 0.2 mL) was added into this Pyrex tube. The Pyrex tube was sealed after being evacuated under vacuum for three pump–thaw cycles in a liquid nitrogen bath. After that, the sealed Pyrex tube was placed in a

vacuum oven at 120 °C for 3 days. The solid was isolated by centrifugation, washed with ethyl acetate, and further purified by Soxhlet extraction with tetrahydrofuran, methanol, and trichloromethane. The sample was then transferred to a vacuum chamber and evacuated to 20 mTorr at 50 °C for 24 h, yielding COFs as yellow powders (yield: 30.0 mg, 94.64%).

**FCTD-TABT**

**FCTD** (19.8 mg, 0.03 mmol) and **TABT** (19.6 mg, 0.03 mmol) were added into a 10 mL Pyrex tube; then, a mixture of o-dichlorobenzene (o-DCB, 1.0 mL), n-butanol (n-BuOH, 1.0 mL), and acetic acid (aq 6 M, 0.2 mL) was added into this Pyrex tube. The Pyrex tube was sealed after being evacuated under vacuum for three pump–thaw cycles in a liquid nitrogen bath. After that, the sealed Pyrex tube was placed in a vacuum oven at 120 °C for 3 days. The solid was isolated by centrifugation, washed with ethyl acetate, and further purified by Soxhlet extraction with tetrahydrofuran, methanol, and trichloromethane. The sample was then transferred to a vacuum chamber and evacuated to 20 mTorr at 50 °C for 24 h, yielding COFs as yellow powders (yield: 29.7 mg, 75.38%).

# 3. Experimental data

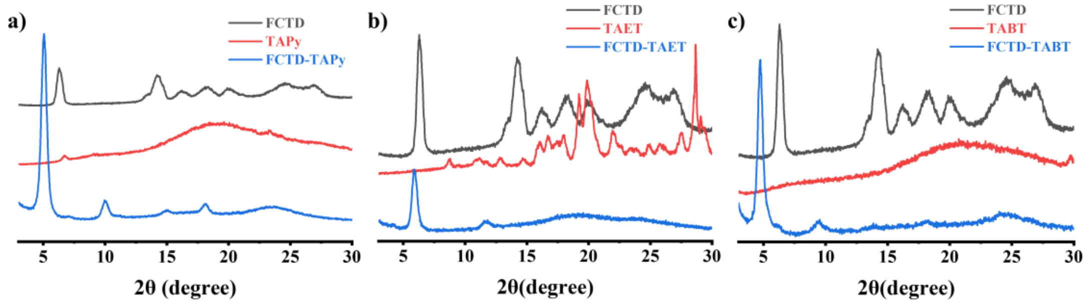


**Figure S1.** PXRD of (a) **FCTD-TAPy**, (b) **FCTD-TAET**, and (c) **FCTD-TABT**

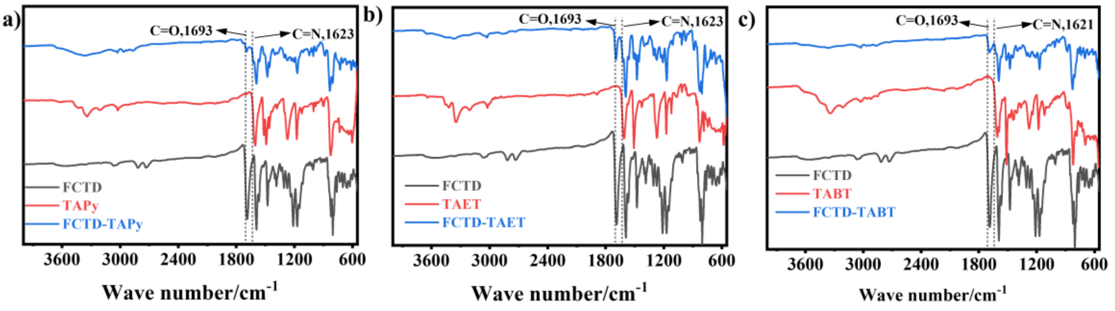


**Figure S2.** FT-IR spectra of (a) **FCTD-TAPy**, (b) **FCTD-TAET**, and (c) **FCTD-TABT**

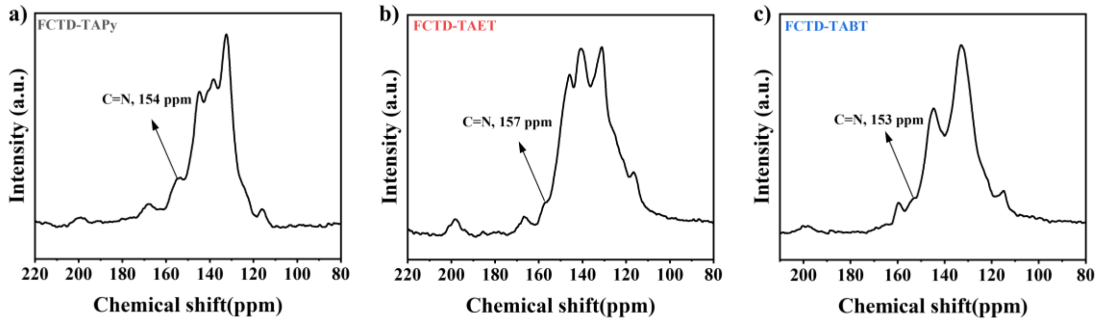


**Figure S3.** $^{13}C$ CP/MAS NMR spectra of (a) **FCTD-TAPy**, (b) **FCTD-TAET**, and (c) **FCTD-TABT**

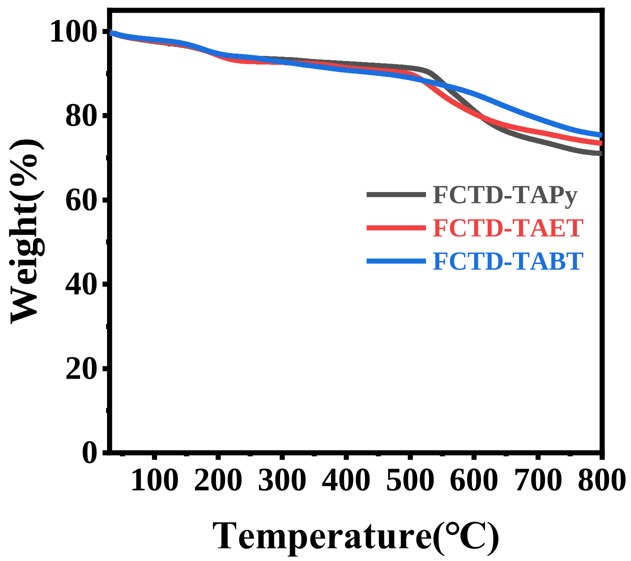


**Figure S4.** Thermogravimetric curves of **FCTD-TAPy**, **FCTD-TAET**, and **FCTD-TABT** under argon atmosphere

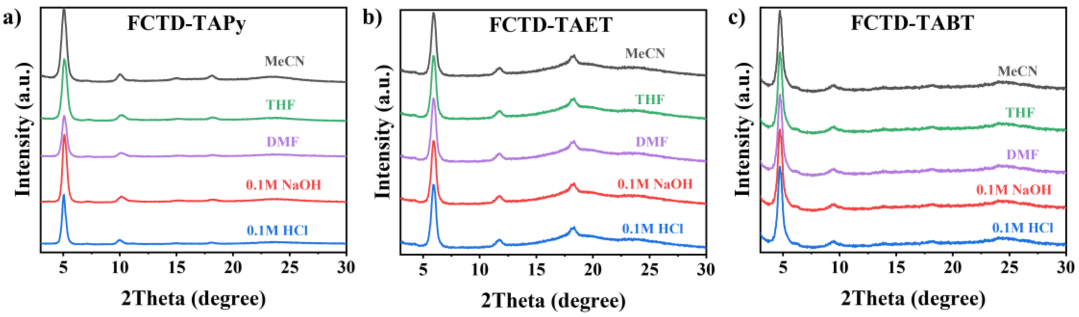


**Figure S5.** The chemical stabilities for (a) **FCTD-TAPy**, (b) **FCTD-TAET**, and (c) **FCTD-TABT** under different solvents.

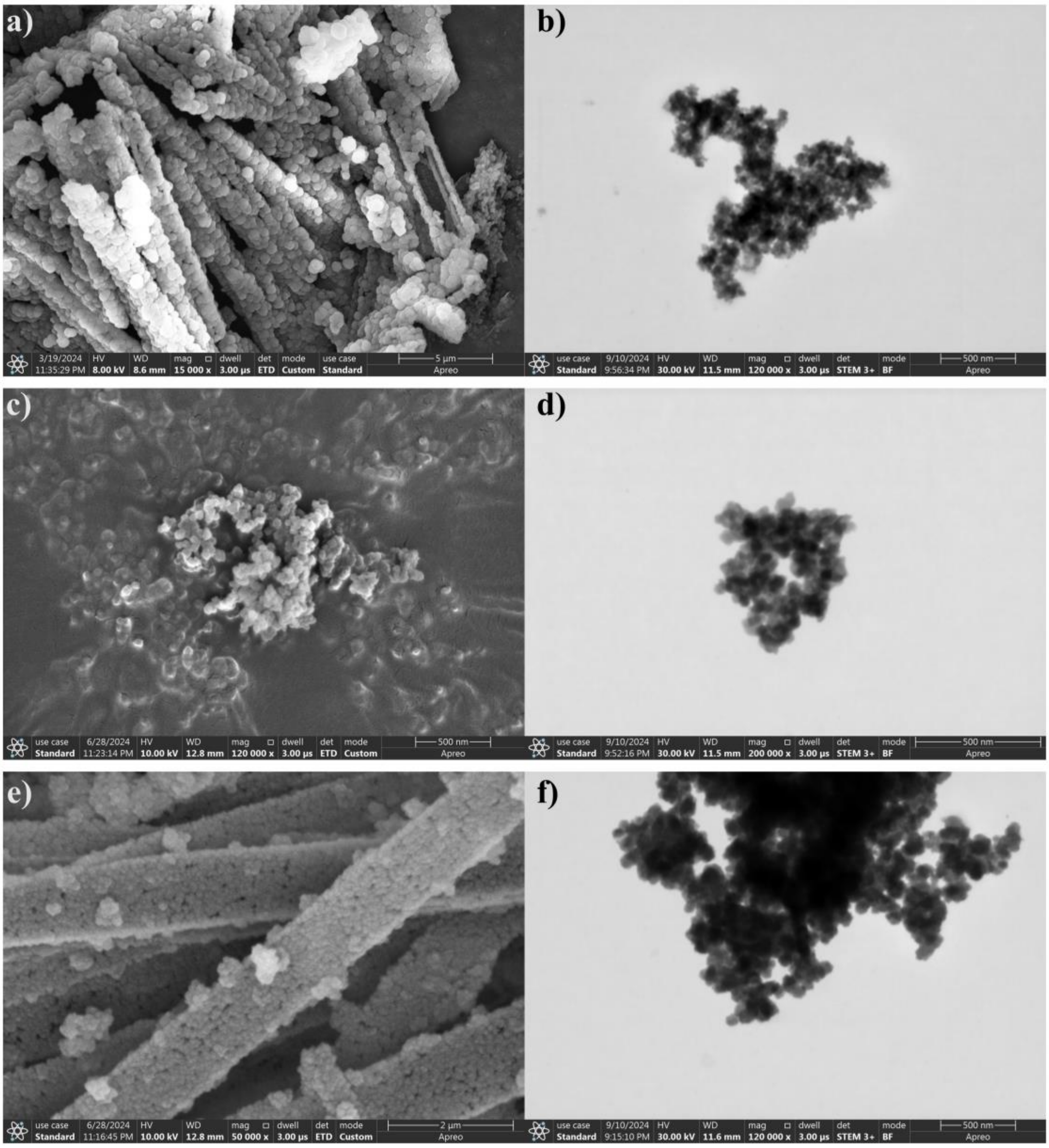


**Figure S6.** The SEM and TEM images of (a-b) **FCTD-TAPy**, (c-d) **FCTD-TAET**, and (e-f) **FCTD-TABT**

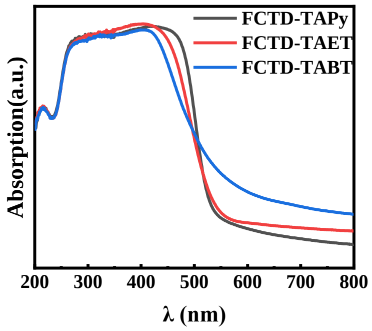


**Figure S7.** UV/visible absorption spectra of the **FCTD-TAPy**, **FCTD-TAET**, and **FCTD-TABT**

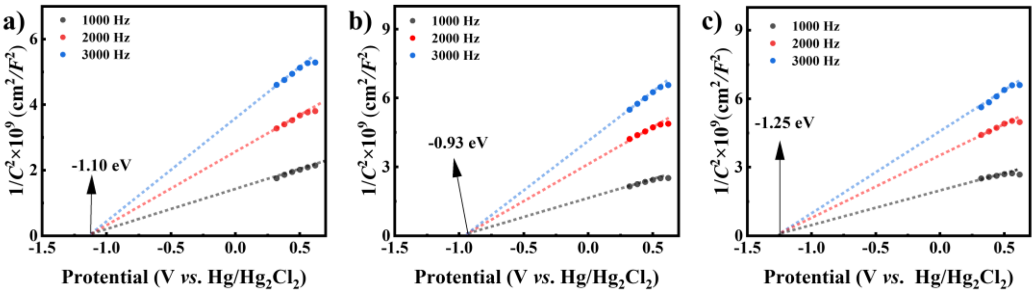


**Figure S8.** Mott-Schottky plots of (a) **FCTD-TAPy**, (b) **FCTD-TAET**, and (c) **FCTD-TABT**

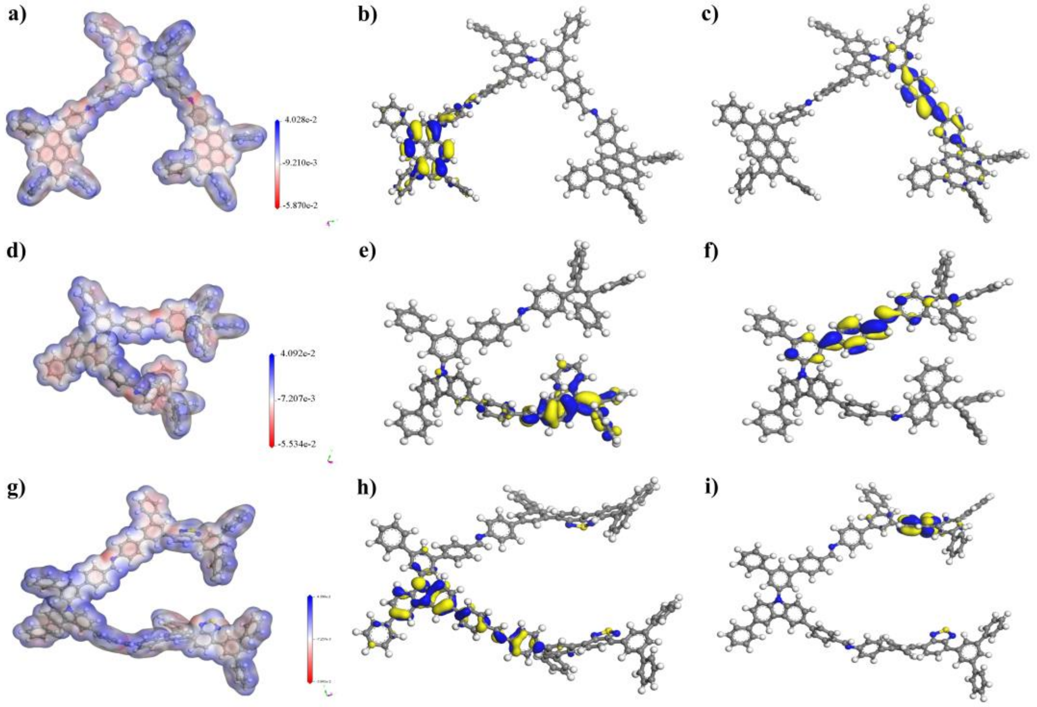


**Figure S9.** (a) Electrostatic potential of **FCTD-TAPy** simple model, (b) HOMO and (c) LUMO molecular orbital diagrams. (d) Electrostatic potential of **FCTD-TAET** simple model, (e) HOMO and (f) LUMO molecular orbital diagrams. (g) Electrostatic potential of **FCTD-TABT** simple model, (h) HOMO and (i) LUMO molecular orbital diagrams.

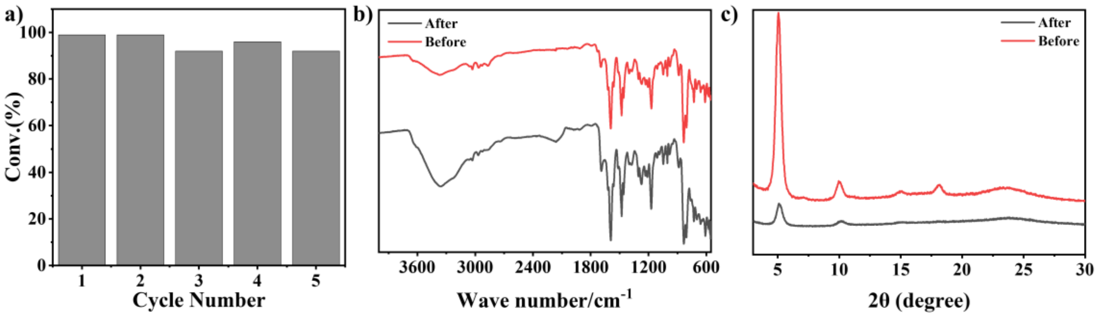


**Figure S10.** (a) Reusability of the **FCTD-TAPy**, (b) PXRD patterns and (c) FT-IR spectra of **FCTD-TAPy** after the photocatalytic reaction.

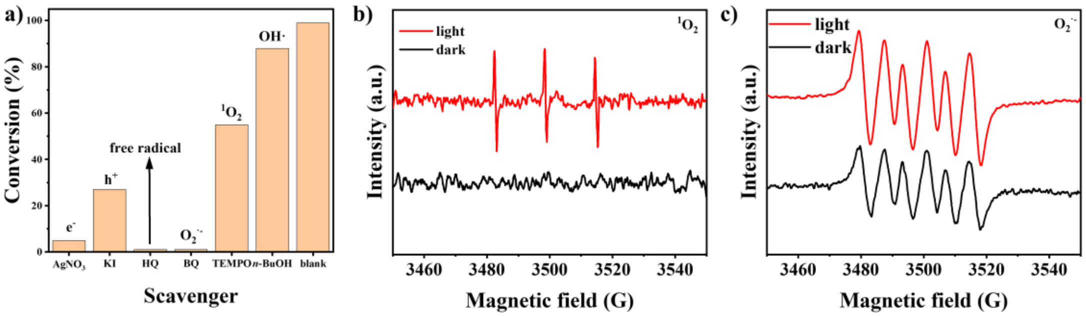


**Figure S11.** (a) Effect of scavengers on the photocatalytic oxidation of benzylamine, (b) in situ EPR signals labeled by TEMPO for ${}^1O_2$ in dispersions and (c) ) in situ EPR signals labeled by DMPO for $O_2^{\bullet-}$ in dispersions.

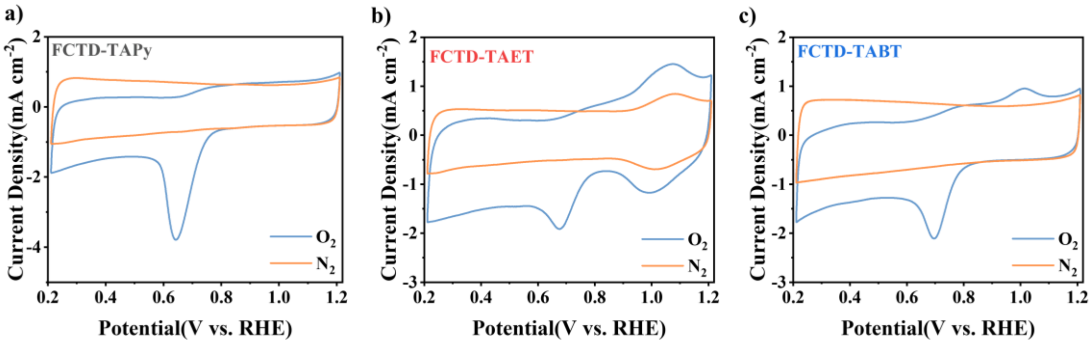


**Figure S12.** CV curves of (a) **FCTD-TAPy**, (b) **FCTD-TAET**, and (c) **FCTD-TABT** at $O_2$ saturation (blue) and $N_2$ saturation (orange)

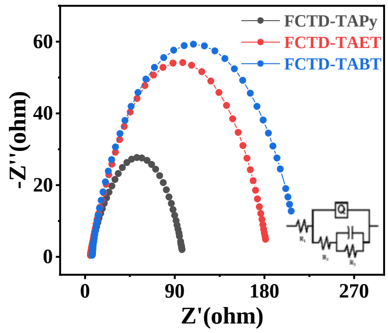

| | $R_1$ | $R_2$ | $R_3$ |
|---|---|---|---|
| **FCTD-TAPy** | 5.26 | 68.97 | 2.43 |
| **FCTD-TAET** | 5.24 | 117.3 | 61.5 |
| **FCTD-TABT** | 6.80 | 1.60 | 207.8 |

**Figure S13.** EIS Nyquist plots of the three COFs

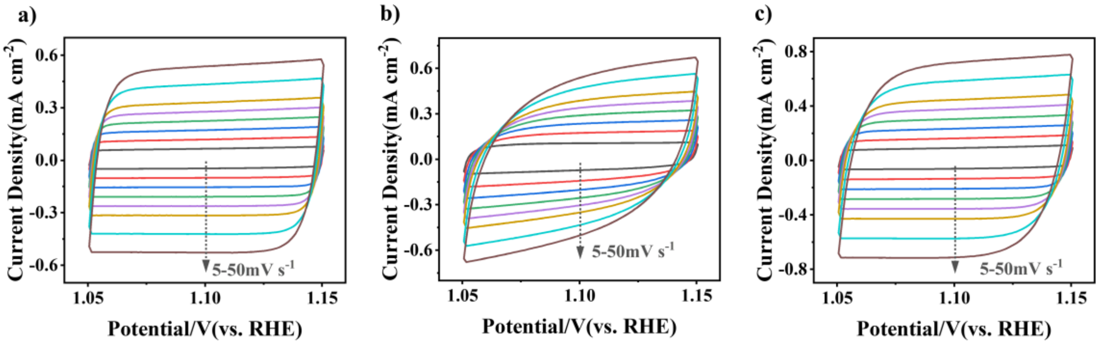


**Figure S14.** CV curves of (a) **FCTD-TAPy**, (b) **FCTD-TAET**, and (c) **FCTD-TABT** at different scanning rates

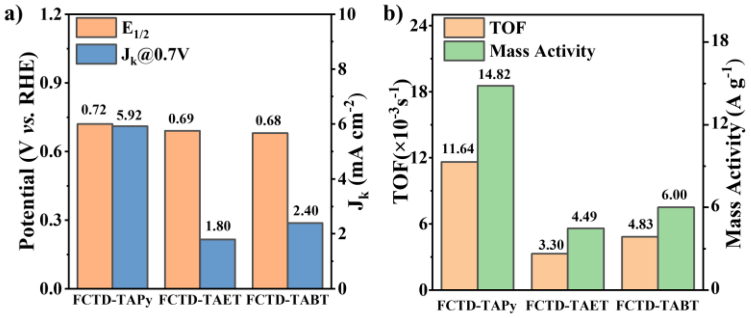


**Figure S15.** (a) $E_{1/2}$ and $J_k$ (0.7V vs. RHE) of three COFs in $O_2$-saturated 0.1 M KOH, (b) TOFs and mass activities (0.7V vs. RHE) of three COFs in $O_2$-saturated 0.1 M KOH.

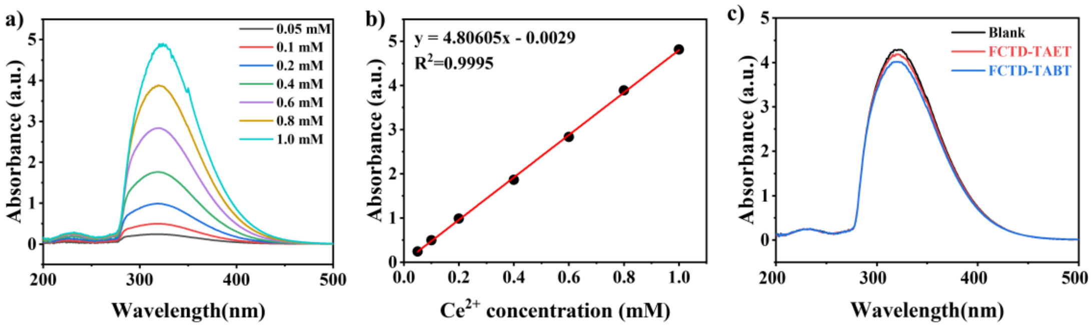


**Figure S16.** UV/visible absorption spectra of $Ce^{4+}$ at different concentrations, (b)The relationship between $Ce^{4+}$concentration at 319 nm and absorbance, (c) Effect of electrolyte on $Ce^{4+}$ absorbance

after electrocatalytic reaction

**Table S1**. Elemental analyses (C, H and N) for COFs

| Sample | Calculated/Found % | | | Calculated | Found |
|---|---|---|---|---|---|
| | C | H | N | C/N | C/N |
| **FCTD-TAPy** | 89.58/88.68 | 4.34/5.56 | 6.08/5.76 | 14.74 | 15.40 |
| **FCTD-TAET** | 88.34/87.04 | 4.99/5.76 | 7.16/7.2 | 12.34 | 12.09 |
| **FCTD-TABT** | 85.30/86.47 | 4.20/6.13 | 7.92/7.4 | 10.77 | 11.69 |

**Table S2.** Influence of different trapping agents on catalytic effect

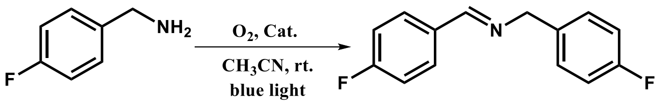


| Entry | Photocatalyst | Blue light | $O_2$ | Time(h) | Quencher | Conv.(%) |
|---|---|---|---|---|---|---|
| **1** | **FCTD-TAPy** | + | + | **12** | **$AgNO_3$** | **5** |
| **2** | **FCTD-TAPy** | + | + | **12** | **KI** | **27** |
| **3** | **FCTD-TAPy** | + | + | **12** | **BQ** | **<1** |
| **4** | **FCTD-TAPy** | + | + | **12** | **TEMPO** | **55** |
| **5** | **FCTD-TAPy** | + | + | **12** | **HQ** | **<1** |
| **6** | **FCTD-TAPy** | + | + | **12** | ***n*-BuOH** | **88** |
| **7** | - | + | + | **12** | / | **17** |
| **8** | **FCTD-TAPy** | - | + | **12** | / | **46** |
| **9** | **FCTD-TAPy** | + | - | **12** | / | **48** |

# 4. Structure simulation data

**Table S3**. Fractional atomic coordinates for simulated **FCTD-TAPy**

| | |
|---|---|
| Space group: P2 | |
| a = 26.740Å, b = 26.150 Å, c = 5.19468 Å | |

| α = γ = 90.0°, β = 118.0° | | | | |
|---|---|---|---|---|
| Atom label | Atom type | x | y | z |
| C1 | C | 0.02755 | 1.95224 | -2.49217 |
| C2 | C | 0.05473 | 1.90502 | -2.48356 |
| C3 | C | 0.05517 | 1.9993 | -2.48565 |
| C4 | C | 0.10965 | 1.90478 | -2.46151 |
| C5 | C | 0.13665 | 1.95136 | -2.44873 |
| C6 | C | 0.11107 | 1.99904 | -2.46076 |
| C7 | C | 0.14148 | 1.8566 | -2.4407 |
| C8 | C | 0.1256 | 1.82482 | -2.68465 |
| C9 | C | 0.15715 | 1.7807 | -2.66676 |
| C10 | C | 0.20543 | 1.76768 | -2.40686 |
| C11 | C | 0.2207 | 1.7991 | -2.16049 |
| C12 | C | 0.18923 | 1.84328 | -2.17842 |
| N13 | N | 0.23705 | 1.72234 | -2.40148 |
| C14 | C | -0.14728 | 2.04633 | -2.58478 |
| C15 | C | -0.15018 | 2.08459 | -2.78094 |
| C16 | C | -0.18966 | 2.12426 | -2.85932 |
| C17 | C | -0.23014 | 2.12596 | -2.76066 |
| C18 | C | -0.22763 | 2.08894 | -2.56004 |
| C19 | C | -0.18593 | 2.04996 | -2.46917 |
| C20 | C | 0.97413 | 1.04496 | 0.50286 |
| C21 | C | 1.02676 | 0.85907 | 0.50829 |
| C22 | C | 0.54647 | 1.33304 | -1.48766 |
| C23 | C | 0.54457 | 1.2791 | -1.51557 |
| C24 | C | 0.67522 | 1.17529 | -1.94387 |
| N25 | N | 0.71938 | 1.15404 | -1.94384 |
| C26 | C | 0.62066 | 1.57822 | -1.7348 |
| C27 | C | 0.62119 | 1.66302 | -1.92962 |
| C28 | C | 0.5916 | 1.62021 | -1.90967 |
| C29 | C | 0.53172 | 1.45279 | -1.58724 |
| C30 | C | 0.521 | 1.5033 | -1.54964 |
| C31 | C | 0.5487 | 1.54454 | -1.59803 |
| C32 | C | 0.58859 | 1.53479 | -1.69645 |
| C33 | C | 0.59685 | 1.4837 | -1.75899 |
| C34 | C | 0.56733 | 1.4427 | -1.71267 |
| C35 | C | 0.71028 | 1.71277 | -1.78569 |
| C36 | C | 0.68057 | 1.66559 | -1.76898 |
| C37 | C | 0.68017 | 1.57992 | -1.5805 |
| C38 | C | 0.70984 | 1.62341 | -1.59505 |
| C39 | C | 0.66121 | 1.18828 | -1.7084 |
| C40 | C | 0.64069 | 1.26651 | -1.51128 |
| C41 | C | 0.67713 | 1.23707 | -1.58059 |

| | | | | |
|---|---|---|---|---|
| C42 | C | 0.58669 | 1.24801 | -1.56902 |
| C43 | C | 0.60933 | 1.16856 | -1.74627 |
| C44 | C | 0.57248 | 1.19788 | -1.68281 |
| H45 | H | 0.17946 | 1.95007 | -2.42018 |
| H46 | H | 0.08912 | 1.83459 | -2.88908 |
| H47 | H | 0.14474 | 1.75686 | -2.85747 |
| H48 | H | 0.25623 | 1.78935 | -1.9531 |
| H49 | H | 0.20182 | 1.86701 | -1.98681 |
| H50 | H | -0.12475 | 2.08213 | -2.8922 |
| H51 | H | -0.19296 | 2.15119 | -3.02495 |
| H52 | H | -0.25991 | 2.08826 | -2.49022 |
| H53 | H | -0.18575 | 2.02109 | -2.31923 |
| H54 | H | 0.95752 | 1.08163 | 0.52119 |
| H55 | H | 1.04631 | 0.82243 | 0.51642 |
| H56 | H | 0.58415 | 1.35282 | -1.45364 |
| H57 | H | 0.64427 | 1.18948 | -2.14857 |
| H58 | H | 0.5975 | 1.69478 | -2.06629 |
| H59 | H | 0.54583 | 1.61985 | -2.02972 |
| H60 | H | 0.54074 | 1.58316 | -1.55221 |
| H61 | H | 0.62491 | 1.47591 | -1.85146 |
| H62 | H | 0.57088 | 1.40516 | -1.78767 |
| H63 | H | 0.68558 | 1.74082 | -1.94956 |
| H64 | H | 0.70343 | 1.54827 | -1.44123 |
| H65 | H | 0.75557 | 1.62441 | -1.46947 |
| H66 | H | 0.65465 | 1.30411 | -1.4197 |
| H67 | H | 0.71546 | 1.25453 | -1.56148 |
| H68 | H | 0.59431 | 1.13228 | -1.85695 |
| H69 | H | 0.53191 | 1.18143 | -1.73066 |
| N70 | N | 0.5 | 1.4175 | 0.5 |
| C71 | C | 0.5 | 1.36126 | 0.5 |
| C72 | C | 0.5 | 1.25355 | 0.5 |

**Table S4**. Fractional atomic coordinates for simulated **FCTD-TAET**

| Space group: P2 | | | | |
|---|---|---|---|---|
| a = 23.040Å, b = 22.610 Å, c = 5.43821 Å<br>α = γ = 90.0°, β = 118.0° | | | | |
| Atom label | Atom type | x | y | z |
| C1 | C | 0.03079 | 1.94395 | -2.48565 |
| C2 | C | 0.07 | 1.88878 | -2.42373 |
| C3 | C | 0.05016 | 1.84086 | -2.60869 |
| C4 | C | 0.09087 | 1.79152 | -2.55266 |
| C5 | C | 0.1518 | 1.78975 | -2.31334 |
| C6 | C | 0.17066 | 1.83675 | -2.12275 |

| | | | | |
|---|---|---|---|---|
| C7 | C | 0.1305 | 1.88629 | -2.1807 |
| N8 | N | 0.19416 | 1.74075 | -2.27814 |
| C9 | C | -0.06339 | 1.99624 | -2.47022 |
| C10 | C | -0.07797 | 2.04791 | -2.63182 |
| C11 | C | -0.12391 | 2.08862 | -2.6298 |
| C12 | C | -0.1551 | 2.07721 | -2.46556 |
| C13 | C | -0.13768 | 2.02758 | -2.29623 |
| C14 | C | -0.09094 | 1.9884 | -2.29131 |
| C15 | C | 0.5553 | 1.32764 | -1.46677 |
| C16 | C | 0.55292 | 1.26614 | -1.49714 |
| C17 | C | 0.76286 | 1.15697 | -1.62438 |
| N18 | N | 0.79316 | 1.11228 | -1.46811 |
| C19 | C | 0.63057 | 1.59862 | -1.79913 |
| C20 | C | 0.63557 | 1.69563 | -1.97805 |
| C21 | C | 0.59927 | 1.65042 | -1.9409 |
| C22 | C | 0.53356 | 1.46171 | -1.59815 |
| C23 | C | 0.52244 | 1.52015 | -1.55927 |
| C24 | C | 0.55259 | 1.5664 | -1.62495 |
| C25 | C | 0.59366 | 1.55217 | -1.74375 |
| C26 | C | 0.60125 | 1.49267 | -1.80205 |
| C27 | C | 0.57028 | 1.44716 | -1.73365 |
| C28 | C | 0.74249 | 1.74051 | -1.87712 |
| C29 | C | 0.70375 | 1.68978 | -1.86986 |
| C30 | C | 0.69829 | 1.59173 | -1.70213 |
| C31 | C | 0.73472 | 1.63709 | -1.73642 |
| C32 | C | 0.70813 | 1.18378 | -1.59568 |
| C33 | C | 0.62739 | 1.26151 | -1.7116 |
| C34 | C | 0.67878 | 1.23566 | -1.74054 |
| C35 | C | 0.60481 | 1.23612 | -1.53572 |
| C36 | C | 0.6843 | 1.15741 | -1.42561 |
| C37 | C | 0.63264 | 1.18319 | -1.39761 |
| H38 | H | 0.00301 | 1.84173 | -2.79305 |
| H39 | H | 0.07617 | 1.755 | -2.6981 |
| H40 | H | 0.21624 | 1.83562 | -1.93059 |
| H41 | H | 0.14705 | 1.92312 | -2.03757 |
| H42 | H | -0.0562 | 2.05541 | -2.76725 |
| H43 | H | -0.13626 | 2.12718 | -2.76204 |
| H44 | H | -0.16255 | 2.01874 | -2.17391 |
| H45 | H | -0.07933 | 1.94932 | -2.16156 |
| H46 | H | 0.60027 | 1.35072 | -1.42084 |
| H47 | H | 0.77592 | 1.17397 | -1.77794 |
| H48 | H | 0.61087 | 1.73592 | -2.08255 |
| H49 | H | 0.54704 | 1.65596 | -2.02061 |

| | | | | |
|---|---|---|---|---|
| H50 | H | 0.54614 | 1.61162 | -1.57746 |
| H51 | H | 0.63008 | 1.48185 | -1.90622 |
| H52 | H | 0.57302 | 1.40264 | -1.79771 |
| H53 | H | 0.71647 | 1.77959 | -1.98955 |
| H54 | H | 0.72324 | 1.5521 | -1.59081 |
| H55 | H | 0.78737 | 1.63191 | -1.65193 |
| H56 | H | 0.60484 | 1.30128 | -1.82832 |
| H57 | H | 0.69569 | 1.2562 | -1.87549 |
| H58 | H | 0.70585 | 1.11682 | -1.31407 |
| H59 | H | 0.61546 | 1.16276 | -1.26334 |
| N60 | N | 0.5 | 1.42247 | 0.5 |
| C61 | C | 0.5 | 1.35905 | 0.5 |
| C62 | C | 0.5 | 1.23492 | 0.5 |

**Table S5**. Fractional atomic coordinates for simulated **FCTD-TABT**

| Space group: P2 | | | | |
|---|---|---|---|---|
| a = 27.4160Å, b = 28.1600 Å, c = 4.4630 Å<br>α = γ = 90.0°, β = 115.0° | | | | |
| Atom label | Atom type | x | y | z |
| C1 | C | 0.09188 | 1.9399 | -2.69083 |
| C2 | C | 0.10582 | 1.89682 | -2.7991 |
| C3 | C | 0.12647 | 1.97863 | -2.64987 |
| C4 | C | 0.15087 | 1.89383 | -2.87126 |
| C5 | C | 0.17738 | 1.93555 | -2.88637 |
| C6 | C | 0.16419 | 1.9785 | -2.78345 |
| C7 | C | 0.17484 | 1.84711 | -2.88157 |
| C8 | C | 0.14288 | 1.80713 | -3.0289 |
| C9 | C | 0.16613 | 1.76182 | -2.97912 |
| C10 | C | 0.22097 | 1.75599 | -2.77732 |
| C11 | C | 0.25345 | 1.79618 | -2.65097 |
| C12 | C | 0.2306 | 1.84122 | -2.70121 |
| N13 | N | 0.24342 | 1.70922 | -2.70151 |
| C14 | C | -0.1882 | 2.0237 | -2.17787 |
| C15 | C | -0.1554 | 2.06417 | -2.05888 |
| C16 | C | -0.17717 | 2.10695 | -2.01427 |
| C17 | C | -0.23195 | 2.10988 | -2.09357 |
| C18 | C | -0.26467 | 2.06957 | -2.2061 |
| C19 | C | -0.24296 | 2.02659 | -2.24683 |
| C20 | C | 0.54846 | 1.35043 | -1.40993 |
| C21 | C | 0.54752 | 1.30102 | -1.43809 |
| C22 | C | 0.7129 | 1.17337 | -1.38039 |
| N23 | N | 0.74378 | 1.15476 | -1.09788 |
| C24 | C | 0.62785 | 1.57143 | -1.6925 |

| | | | | |
|---|---|---|---|---|
| C25 | C | 0.63606 | 1.65094 | -1.87352 |
| C26 | C | 0.60498 | 1.61088 | -1.89459 |
| C27 | C | 0.53416 | 1.45834 | -1.57895 |
| C28 | C | 0.52198 | 1.50505 | -1.54851 |
| C29 | C | 0.54982 | 1.54264 | -1.60337 |
| C30 | C | 0.5933 | 1.53231 | -1.68048 |
| C31 | C | 0.60478 | 1.4847 | -1.72773 |
| C32 | C | 0.57392 | 1.44738 | -1.68757 |
| C33 | C | 0.71834 | 1.6984 | -1.58468 |
| C34 | C | 0.68964 | 1.65305 | -1.63732 |
| C35 | C | 0.68234 | 1.5724 | -1.46911 |
| C36 | C | 0.71293 | 1.61335 | -1.43796 |
| C37 | C | 0.67509 | 1.21121 | -1.40286 |
| C38 | C | 0.64541 | 1.27763 | -1.17317 |
| C39 | C | 0.68595 | 1.2458 | -1.1558 |
| C40 | C | 0.5936 | 1.27349 | -1.43058 |
| C41 | C | 0.62447 | 1.20939 | -1.66865 |
| C42 | C | 0.58412 | 1.23987 | -1.68037 |
| C43 | C | 0.95582 | 0.94326 | 0.60312 |
| C44 | C | 0.97941 | 0.98651 | 0.56426 |
| C45 | C | 1.02153 | 0.90131 | 0.4558 |
| N46 | N | 0.96913 | 1.03042 | 0.63292 |
| H47 | H | 0.08353 | 1.86469 | -2.81965 |
| H48 | H | 0.12634 | 2.00904 | -2.50542 |
| H49 | H | 0.20857 | 1.93425 | -2.97472 |
| H50 | H | 0.09994 | 1.8107 | -3.17305 |
| H51 | H | 0.14129 | 1.73094 | -3.08441 |
| H52 | H | 0.29638 | 1.79258 | -2.50698 |
| H53 | H | 0.25625 | 1.87119 | -2.58252 |
| H54 | H | -0.11281 | 2.06255 | -1.99716 |
| H55 | H | -0.1521 | 2.13824 | -1.92955 |
| H56 | H | -0.30728 | 2.07215 | -2.26947 |
| H57 | H | -0.26906 | 1.99607 | -2.33984 |
| H58 | H | 0.58646 | 1.36879 | -1.32585 |
| H59 | H | 0.70758 | 1.15556 | -1.60859 |
| H60 | H | 0.61738 | 1.68138 | -2.02783 |
| H61 | H | 0.56257 | 1.61123 | -2.05928 |
| H62 | H | 0.53919 | 1.57885 | -1.57713 |
| H63 | H | 0.63689 | 1.4768 | -1.8009 |
| H64 | H | 0.58073 | 1.41159 | -1.74675 |
| H65 | H | 0.70365 | 1.72438 | -1.78269 |
| H66 | H | 0.70008 | 1.54263 | -1.30532 |
| H67 | H | 0.75417 | 1.61474 | -1.25265 |

| | | | | |
|---|---|---|---|---|
| H68 | H | 0.65335 | 1.30312 | -0.97627 |
| H69 | H | 0.72504 | 1.24708 | -0.94706 |
| H70 | H | 0.61547 | 1.18313 | -1.86242 |
| H71 | H | 0.54496 | 1.23654 | -1.88316 |
| H72 | H | 1.03692 | 0.86677 | 0.44015 |
| S73 | S | 1 | 1.07692 | 0.5 |
| N74 | N | 0.5 | 1.42671 | 0.5 |
| C75 | C | 0.5 | 1.37572 | 0.5 |
| C76 | C | 0.5 | 1.27685 | 0.5 |

# 5. Others

**Table S6.** Different COFs performance for electrocatalytic ORR

| Materials | $E_o$ (V) | $E_{1/2}$ (V) | Tafel slope (mV/dec) | $C_{dl}$ (mF cm$^{-2}$) | Ref |
|---|---|---|---|---|---|
| FCTD-TAPy | 0.84 | 0.72 | 51.07 | 10.56 | This work |
| FCTD-TAET | 0.84 | 0.69 | 66.55 | 9.51 | |
| FCTD-TABT | 0.87 | 0.68 | 53.01 | 14.26 | |
| JUC-616 | / | 0.78 | 52.91 | 8.62 | [1] |
| CoPc-2$SO_3$H-COF | 0.82 | / | 63.2 | 0.15 | [2] |
| COF-112Co | 0.87 | 0.76 | 74.32 | 9.11 | [3] |
| CoF/NG | 0.91 | 0.80 | 78 | 9.66 | [4] |
| Py-TD-COF | 0.834 | 0.698 | 252 | / | [5] |
| Py-TD-COF-NH | 0.829 | 0.693 | 281 | / | |
| PTh-COF/CNT | / | 0.75 | 45.7 | 8.6 | [6] |
| PYTA-PB-COF | 0.88 | 0.78 | 67.9 | 7.9 | [7] |

| | | | | | |
|---|---|---|---|---|---|
| CoPc-EA-COF | 0.901 | 0.803 | 128 | 16.2 | [8] |
| CoNP-s-IMCOF | / | 0.83 | 107 | 0.031 | [9] |
| CoTAPP-PATA-COF | 0.926 | 0.801 | 55 | 10.64 | [10] |

**Table S7.** Comparisons of different photocatalysts for the selective oxidation of benzylamine

| Photocatalyst | Conditions | Conv.(%) | Sel.(%) | Ref |
|---|---|---|---|---|
| COF-TpPa (10 mg) | Benzylamine (0.9 mmol), $O_2$ (1 atm), $CH_3CN$ (6 mL), 5.0 W 420 nm LED, and 5.0 h | 78 | 99 | [11] |
| ToAzo-COF (5 mg) | Benzylamine (0.3 mmol), Air, $CH_3CN$ (1 mL), rt., 3.0 W × 4 460 nm LED, and 1.8 h | 90 | 99 | [12] |
| BTT-Bpy-COF (10 mg) | Benzylamine (0.5 mmol), $O_2$ (1 atm), $CH_3CN$ (3 mL), rt., 5.0 W × 4 465 nm LED, and 3 h | 99 | 99 | [13] |
| W18O49@TpPa-H-0,1 (10 mg) | Benzylamine (0.1 mmol), Air , $CH_3CN$ (5 mL), rt., 10.0 W 420-425 nm LED, and 2 h | 99 | 99 | [14] |
| UiO-66-$NH_2$@$Au_{0.5}$@COF1 (20 mg) | Benzylamine (1 mmol), $O_2$ (1 atm), $CH_3CN$ (3 mL), 420 nm light, and 20 h | 66.9 | 96.9 | [15] |
| COF-NUST-36 (4 mg) | Benzylamine (0.1 mmol), $O_2$ (1 atm), $CH_3CN$ (1.5 mL), rt.,30.0 W 455-460 nm light, and 2.5 h | 97 | 95 | [16] |
| PY-BT COF (5 mg) | Benzylamine (0.1 mmol), $O_2$ (1 atm), $CH_3CN$ (2 mL), rt.,300 W 455-460 nm light, and 2.5 h | 99 | / | [17] |
| FCTD-TAPy (5 mg) | 4-Methylbenzylamine (0.1 mmol), $O_2$ (1 atm), $CH_3CN$ (2 mL), rt.,30 W 450-460 nm light, and 12 h | 95 | 99 | This work |

# 6. References


1. J. Li, J. Jia, J. Suo, C. Li, Z. Wang, H. Li, V. Valtchev, S. Qiu, X. Liu, Q. Fang. *Journal of Materials Chemistry A*, 2023, **11**, 18349.
2. R. Jiang, Q. Zhi, B. Han, N. Li, K. Wang, D. Qi, W. Li, J. Jiang. *Chemical Engineering Journal*, 2024, **489**, 151232.
3. J. Li, P. Liu, J. Mao, J. Yan, W. Song. *Nanoscale*, 2022, **14**, 6126.
4. Y. Shin, S. Park, H. Jang, G. Shin, D. Shin, S. Park. *Nanoscale*, 2024.
5. S. Huang, B. Zhang, D. Wu, Y. Xu, H. Hu, F. Duan, H. Zhu, M. Du, S. Lu. *Applied Catalysis B: Environmental*, 2024, **340**, 123216.
6. Z. Chen, P. Fang, X. Zou, Z. Shi, J. Zhang, Z. Sun, S. Guo, F. Yan. *Small*, 2024, 2401880.
7. X. Yang, Y. Fu, M. Liu, S. Zheng, X. Li, Q. Xu, G. Zeng. *Angewandte Chemie International Edition*, 2024, **63**, e202319247.
8. M. Liu, S. Yang, Y. Fu, X. Yang, X. Li, J. He, Q. Xu, G. Zeng. *Chemical Engineering Journal*, 2024, **488**, 150812.
9. J.-M. Ju, C. H. Lee, J. H. Park, J.-H. Lee, H. Lee, J.-H. Shin, S.-Y. Kwak, S. U. Lee, J.-H. Kim. *ACS applied materials & interfaces*, 2022, **14**, 24404.
10. M. Liu, S. Liu, C. X. Cui, Q. Miao, Y. He, X. Li, Q. Xu, G. Zeng. *Angewandte Chemie*, 2022, **134**, e202213522.
11. Z. Wu, X. Huang, X. Li, G. Hai, B. Li, G. Wang. *Science China Chemistry*, 2021, **64**, 2169.
12. K. Xiong, Y. Wang, F. Huang, K. Zhang, B. Zeng, X. Lang. *Journal of Colloid and Interface Science*, 2024, **665**, 252.
13. J. Kou, G. Wang, H. Guo, L. Li, J. Fang, J. Ma, Z. Dong. *Applied Catalysis B: Environment and Energy*, 2024, **352**, 124020.
14. X. Zhao, A. Li, D. Yang, T.-Y. Qiu, Z. Zhao, S.-L. Wang, X. Mu, H.-Q. Tan. *Journal of Colloid and Interface Science*, 2024, **653**, 67.
15. K. Zhang, Z. Xi, Z. Wu, G. Lu, X. Huang. *ACS Sustainable Chemistry & Engineering*, 2021, **9**, 12623.
16. Z. Gu, Z. Shan, Y. Wang, J. Wang, T. Liu, X. Li, Z. Yu, J. Su, G. Zhang. *Chinese Chemical Letters*, 2024, **35**, 108356.
17. M.-Y. Yang, S.-B. Zhang, M. Zhang, Z.-H. Li, Y.-F. Liu, X. Liao, M. Lu, S.-L. Li, Y.-Q. Lan. *Journal of the American Chemical Society*, 2024, **146**, 3396.
